# Myocardial structural and functional changes in patients with liver cirrhosis awaiting liver transplantation: a comprehensive cardiovascular magnetic resonance and echocardiographic study

**DOI:** 10.1186/s12968-020-00622-2

**Published:** 2020-04-23

**Authors:** Hyue Mee Kim, Hyung-Kwan Kim, Jeong-Hoon Lee, Yun Bin Lee, Eun-Ah Park, Jun-Bean Park, Seung-Pyo Lee, Yoon Jun Kim, Yong-Jin Kim, Jung-Hwan Yoon, Dae-Won Sohn

**Affiliations:** 1 Division of Cardiology, Department of Internal Medicine and Cardiovascular Center, Section of Cardiovascular Imaging, Seoul National University Hospital, 103 Daehak-ro, Jongno-gu, Seoul, 03080 South Korea; 2 Division of Cardiology, Department of Internal Medicine, Chung-Ang University Hospital, Seoul, South Korea; 3 Division of Gastroenterology, Department of Internal Medicine and Liver Research Institute, Seoul National University College of Medicine, 103 Daehak-ro, Jongno-gu, Seoul, 03080 South Korea; 4 Department of Radiology, Seoul National University Hospital, Seoul, South Korea

**Keywords:** Extracellular volume fraction, Cardiovascular magnetic resonance, Left ventricle, Global longitudinal strain, Cirrhosis

## Abstract

**Background:**

Cardiac dysfunction is increasingly recognized in patients with liver cirrhosis. Nevertheless, the presence or absence of structural alterations such as diffuse myocardial fibrosis remains unclear. We aimed to investigate myocardial structural changes in cirrhosis, and explore left ventricular (LV) structural and functional changes induced by liver transplantation.

**Methods:**

This study included 33 cirrhosis patients listed for transplantation and 20 healthy controls. Patients underwent speckle-tracking echocardiography and cardiovascular magnetic resonance (CMR) with extracellular volume fraction (ECV) quantification at baseline (*n* = 33) and 1 year after transplantation (*n* = 19).

**Results:**

CMR-based LV ejection fraction (CMR_LV-EF_) and echocardiographic LV global longitudinal strain (LV-GLS) demonstrated hyper-contractile LV in cirrhosis patients (CMR_LV-EF_: 67.8 ± 6.9% in cirrhosis vs 63.4 ± 6.4% in healthy controls, *P* = 0.027; echocardiographic GLS: − 24.2 ± 2.7% in cirrhosis vs − 18.6 ± 2.2% in healthy controls, *P* < 0.001). No significant differences in LV size, wall thickness, mass index, and diastolic function between cirrhosis patients and healthy controls were seen (all *P* > 0.1). Only one of the cirrhosis patients showed late gadolinium enhancement. However, cirrhosis patients showed a higher ECV (31.6 ± 5.1% vs 25.4 ± 1.9%, *P* < 0.001) than healthy controls. ECV showed a positive correlation with Child-Pugh score (*r* = 0.564, *P* = 0.001). Electrocardiogram-based corrected QT interval was prolonged in cirrhosis (*P* < 0.001). One-year post-transplantation, echocardiographic LV-GLS (from − 24.9 ± 2.4% to − 20.6 ± 3.4%, *P* < 0.001) and ECV (from 30.9 ± 4.5% to 25.4 ± 2.6%, *P* = 0.001) moved to the normal ranges. Corrected QT interval decreased after transplantation (from 475 ± 41 to 429 ± 30 msec, *P* = 0.001).

**Conclusions:**

Myocardial extracellular volume expansion with augmented resting LV systolic function was characteristic of cirrhotic cardiomyopathy, which normalizes 1-year post-transplantation. Thus, myocardial extracellular expansion represents a structural component of myocardial changes in cirrhosis.

## Background

Liver cirrhosis is associated with chronic dysfunction in the cardiovascular system [1–4]. Cardiac dysfunction is usually subclinical at rest, but can be clinically manifest, especially when rapid blood volume shift occurs, for example in transjugular intrahepatic portosystemic shunt or liver transplantation, which subsequently contributes to cardiovascular complications [5–7]. This cardiac condition in cirrhosis is referred to as cirrhotic cardiomyopathy. This unique cardiomyopathy is characterized by blunted contractile response to stress stimuli, diastolic dysfunction, and electrophysiological abnormalities in the absence of gross cardiac diseases [1–4]. Since myocardial changes observed in cirrhosis were mostly based on echocardiography, earlier studies mainly focused on functional and hemodynamic changes without co-demonstration of anatomical changes. Thus, myocardial structural changes in cirrhosis remain largely unclear, although diffuse myocardial fibrosis (DMF) was suggested, mostly in patients with alcoholic cirrhosis [8, 9]. Therefore, whether functional changes on echocardiography are secondary to myocardial structural changes, or are simply collateral phenomena resulting from cirrhosis -induced hemodynamic alteration without myocardial structural changes remains to be established.

Cardiovascular magnetic resonance (CMR) with late gadolinium enhancement (LGE) is a widely utilized non-invasive imaging protocol for myocardial tissue characterization. However, a drawback of LGE is its insufficient sensitivity in detecting reversible, early stage diffuse interstitial fibrosis or DMF [10, 11]. Recently, extracellular volume fraction (ECV) quantification by CMR T1 mapping technique has been introduced to evaluate DMF in vivo. Previous studies demonstrated that ECV reliably reflect the degree of DMF and, more importantly, is associated with prognosis in various cardiac diseases [12–14]. Thus, in this study, we hypothesized that myocardium in liver cirrhosis has structural alterations such as DMF, which could be effectively addressed by CMR. We also attempted to relate the structural changes to functional or hemodynamic changes on echocardiography. Finally, we evaluated whether these changes could be reversed by liver transplantation.

## Methods

### Study population

From March 2016 to December 2017, 36 patients with liver cirrhosis of various etiologies who were listed on the transplant waitlist were prospectively enrolled, of whom three (8.3%) were excluded as they failed to undergo CMR due to acute clinical deterioration. All included patients had been referred to the cardiology department for cardiac evaluation before transplantation. Liver cirrhosis was confirmed based on clinical, laboratory, and ultrasonographic findings [15–17]. Patients with any of the following were systematically excluded: aged < 18 years, decreased kidney function (estimated glomerular filtration rate < 30 mL/min/1.73 m^2^), documented history of cardiovascular diseases including coronary artery disease, and other forms of myocardial disease, and acute liver failure without cirrhosis. All patients underwent computed tomographic coronary angiography or invasive coronary angiography as a routine preoperative assessment. The absence of a significant coronary artery disease (> 50% luminal stenosis) was verified. Electrocardiogram (ECG), transthoracic echocardiography, and CMR were performed at baseline (*n* = 33) and repeated at 1 year after transplant (*n* = 19). Clinical data on baseline characteristics were collected. Liver cirrhosis severity was categorised according to the Child-Pugh score. For comparison, 20 subjects without any cardiovascular disease or cardiovascular disease risk factors were included as healthy controls. This study was conducted according to the principles of the Declaration of Helsinki and approved by the Institutional Review Board of our institution. Written informed consent was obtained from all participants.

### Transthoracic echocardiography and ECG

Echocardiographic and ECG examinations were conducted as part of the preoperative cardiac evaluation. Echocardiographic data were obtained with commercially available equipments (Vivid 7, General Electric Healthcare, Horten, Norway; E9, Philips Healthcare, Andover, Massachusetts, USA; Acuson SC2000, Siemens Medical Solutions, Mountain View, California, USA). Conventional echocardiographic parameters were measured by experienced sonographers. Left ventricular (LV) ejection fraction (LVEF) was calculated in accordance with the American Society of Echocardiography guideline [18]. To assess LV diastolic function, the transmitral inflow peak early (E) and late (A) diastolic velocities, early septal mitral annular diastolic velocity (e’), left atrial (LA) volume index (LAVI), and peak tricuspid regurgitation velocity were recorded. LA volumes were measured using biplane area-length method at the end-ventricular systole, and LA volume index (LAVI) was calculated as LA volume divided by body surface area. LV diastolic function was graded based on the 2016 recommendation [19]. Contrast echocardiography using agitated saline was performed through a left antecubital intravenous line to assess the presence or absence of intrapulmonary shunt. Intrapulmonary shunt was confirmed when bubbles were visualized in the LA and/or LV > 3 beats after its appearance in the right atrium. Data on the LV global longitudinal strain (GLS) and global circumferential strain (GCS) were acquired using two-dimensional speckle-tracking echocardiography based on the guideline [20, 21]. Speckle-tracking echocardiography was used to assess strain values because it has been already incorporated into the daily clinical practice and is now the widely available technique of choice for the assessment of LV strain [20, 21]. LV GLS and GCS measurement and analyses were performed using commercially available software (TomTec, Image Arena 4.6, Munich, Germany).

On ECG QT interval was measured from the start of the Q wave to the end of the T-wave in lead II. To adjust for the heart rate, corrected QT (QTc) interval was calculated by dividing the QT interval by the square root of the R-R interval.

### CMR

CMR was performed using a 3 T CMR system (MAGNETOM Skyra; Siemens Healthineers, Erlangen, Germany) equipped with six-channel phased-array coils [22]. After acquiring scout images for localization, balanced steady-state free precession cine images were obtained during breath-holding. To include the entire LV volume, LV short-axis images (6-mm thickness with 4-mm intersection gap) were acquired at 10-mm intervals from the base to apex using retrospective ECG gating with the following parameters: repetition time /echo time, 2.8–3.2 msec/1.4–1.6 msec; flip angle, 80°; temporal resolution, 42 msec; field of view, 240 × 300 mm; and matrix, 256 × 150. LV end-diastolic volume (EDV), end-systolic volume (ESV) volumes, and LVEF were measured from the cine images. LV stroke volume, cardiac index and mass index were subsequently calculated.

ECV calculation was performed as follows. After acquiring cine images, a mid-ventricular short-axis section at the papillary muscle level was obtained using the modified Look-Locker inversion-recovery sequence, with three images in the first two Look-Locker segments and five images for the third inversion (the “3–3-5” standard protocol) [23]. Finally, 11 images acquired in 17 heartbeats were obtained, and in-line motion correction and map generation were performed. The following readout parameters were used: section thickness, 6 mm; 2.5/1.1; 6/8 partial Fourier acquisition; field-of-view, 240 × 300 mm; and matrix, 192 × 125. Region of interest was drawn manually at the mid-ventricular septum and LV blood pool on pre- and post-contrast T1 mapping images to measure the ECV. Care was taken to draw the region-of-interest on the compact myocardium and to not include the border of the myocardium because it shows gradual changes in T1 values due to both partial volume averaging artifact and registration error, even after motion correction. One radiologist (E-A Park, with > 10 years of CMR experience) performed all measurements, and myocardial T1 could be measured reliably with one large region of interest on all images. Intra- and inter-observer variability was previously reported at our institution for this measurement [12]. ECV was estimated as follows: ECV = (1 – hematocrit) × [1/T1 _myocardium post_ - 1/T1_myocardium pre_]/[1/T1 _blood post_ - 1/T1_blood pre_] [24].

To evaluate irreversible myocardial fibrosis, the presence or absence of LGE was assessed. LGE images were acquired 10 min after intravenous gadolinium administration (0.2 mmol/kg Magnevist; Schering, Berlin, Germany), immediately followed by 20 mL saline flush. The protocol for obtaining LGE images was as follows: slice thickness, 6 mm; interslice gap, 4 mm; TR, 9.1 msec; TE, 42 msec; flip angle, 13 degrees; and in-plane resolution, 1.4 × 1.9 mm. LGE images from the same image planes as those in the cine images were acquired using inversion recovery segmented spoiled-gradient echo and phase-sensitive inversion recovery sequences. The most appropriate inversion time was set to null the normal myocardium, which was typically between 250 and 300 msec, in both pre- and post-transplant CMR examinations. LGE CMR images were analyzed by an experienced radiologist (E-A Park), who was blinded to the patients’ information.

At the last stage of this study, myocardial T2 mapping data was acquired in 6 patients using adiabatic T2-prepared fast low-angle shot (FLASH) technique. Three single-shot FLASH images with different T2 preparation times were acquired as follow: echo time (ms): 0, 30, 55 respectively; slice thickness 6 mm; TR, 2.4 msec; TE, 1.0 msec; flip angle, 12 degrees; receiver bandwidth 1184 Hz/pixel; field of view, 288 × 360 mm; and matrix, 144 × 192.

### Statistical analysis

Baseline characteristics are described as number and percentages for categorical variables, and mean ± standard deviation for continuous variables. Patient characteristics were compared between groups using a chi-square or Fisher’s exact test for categorical variables, and Student’s t test or Mann-Whitney U test for continuous variables, as appropriate. Changes in echocardiographic, ECG, and CMR parameters between pre- and post-transplant were compared using paired t-test or Wilcoxon signed rank test. Pearson’s correlation coefficient was used to evaluate the relationship between the relevant parameters and Child-Pugh score. A partial correlation analysis between ECV and Child-Pugh score was performed while controlling for cardiac index. All statistical analyses were performed using SPSS (v22.0 Statistical Package for the Social Sciences, International Business Machines, Inc., Armonk, New York, USA). A *P*-value < 0.05 was considered statistically significant.

## Results

### Baseline characteristics

A total of 33 patients were analyzed in the final analysis. The patients’ baseline characteristics are summarized in Table 1. Mean age of cirrhosis patients was 56.3 ± 9.9 years and 25 (75.8%) were men. The etiology of cirrhosis was viral in 20 patients (60.6%), alcoholic in 9 (27.3%), and autoimmune hepatitis in 2 (6.1%). Most patients (*n* = 23, 69.7%) were in Child-Pugh class C and their heart rate was higher than that of patients with Child-Pugh class A/B (*P* = 0.002). There were no significant differences in the prevalence of hypertension and diabetes mellitus and in the levels of hemoglobin and creatinine between the Child-Pugh class A/B and Child-Pugh class C groups. No significant difference in cardiovascular medications at the time of CMR except for beta-blockers was noted.

**Table 1:** Baseline clinical characteristics of 33 patients with liver cirrhosis

	All	Child-Pugh class A/B	Child-Pugh class C	***P*** value
	***N*** = 33	***N*** = 10	***N*** = 23	
Age (years)	56.3 ± 9.9	58.8 ± 7.3	55.3 ± 10.8	0.355
Male (n, %)	25 (75.8%)	9 (90.0%)	16 (69.6%)	0.208
Systolic blood pressure (mmHg)	113.47 ± 14.0	111.5 ± 7.6	114.4 ± 16.2	0.601
Diastolic blood pressure (mmHg)	66.3 ± 12.3	60.9 ± 9.0	68.8 ± 12.9	0.094
Heart rate (/min)	73.6 ± 15.7	63.3 ± 9.1	78.0 ± 16.0	0.002
**Cirrhosis etiology (n, %)**				0.296
Viral	20 (60.6%)	8 (80.0%)	12 (52.2%)	
HBV	17 (51.5%)	6 (60.0%)	11 (47.8%)	
HCV	3 (9.1%)	2 (20.0%)	1 (4.3%)	
Alcoholic	9 (27.3%)	1 (10.0%)	8 (34.8%)	
Autoimmune hepatitis	2 (6.1%)	0 (0%)	2 (8.7%)	
Cryptogenic	2 (6.1%)	1 (10.0%)	1 (4.3%)	
Child-Pugh score	9.8 ± 2.4	6.8 ± 1.3	11.1 ± 1.2	< 0.001
MELD score	18.8 ± 7.4	11.1 ± 2.1	22.1 ± 6.3	< 0.001
**Underlying diseases (n, %)**				
Hypertension	8 (24.2%)	4 (40.0%)	4 (17.4%)	0.164
Diabetes mellitus	9 (27.3%)	4 (40.0%)	5 (21.7%)	0.279
**Medication (n, %)**				
Beta-blockers	8 (24.2%)	6 (60.0%)	2 (8.7%)	0.002
Diuretics	17 (51.5%)	4 (40.0%)	13 (56.5%)	0.383
ACEI/ARB	3 (9.1%)	2 (20%)	1 (4.3%)	0.151
**Laboratory examination**				
Hemoglobin (g/dL)	10.6 ± 1.6	11.4 ± 1.6	10.2 ± 1.5	0.050
Creatinine (mg/dL)	0.9 ± 0.4	0.8 ± 0.2	0.9 ± 0.4	0.565
Bilirubin (mg/dL)	7.5 ± 8.5	1.7 ± 0.6	10.0 ± 9.1	< 0.001
Albumin (g/dL)	2.9 ± 0.4	3.2 ± 0.4	2.7 ± 0.4	0.002
PT (INR)	1.9 ± 1.0	1.3 ± 0.2	2.1 ± 1.1	0.001

Values are shown as number (%) or mean ± standard deviation

*HBV* hepatitis B virus, *HCV* hepatitis C virus, *MELD* the model for end stage liver disease, *ACEI* angiotensin-converting enzyme inhibitors, *ARB* angiotensin II receptor blockers, *PT* prothrombin time, *INR* international normalized ratio

### Echocardiographic and ECG parameters in cirrhosis

**Fig. 1:**
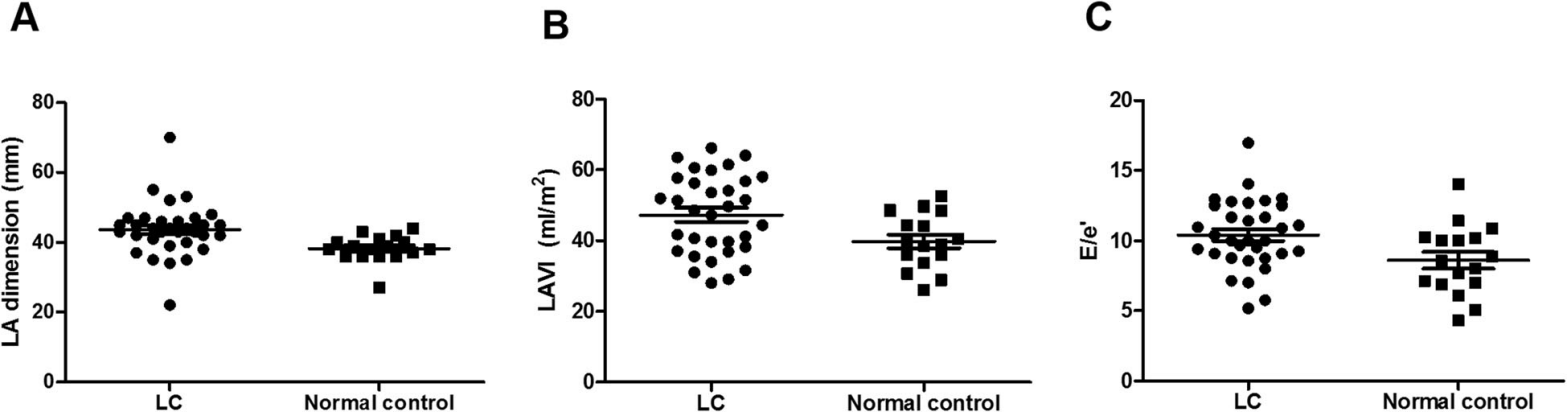
Left ventricular diastolic functional parameters in patients with cirrhosis and normal controls. LA, left atrium; LAVI, left atrial volume index

Comparisons of echocardiographic and ECG parameters are shown in Table 2. No significant differences in LV size, LV wall thickness, and Doppler transmitral inflow patterns were found between the healthy controls (65.0 ± 14.8 years; men, 11 (55%)) and cirrhosis patients were observed. Notably, LVEF, a conventional index for LV systolic function, was significantly higher in cirrhosis patients than in normal controls (*P* = 0.049), indicating a hyper-contractile state in cirrhosis. Resting hyper-contractile state in cirrhosis was corroborated by a significant augmentation in GLS (− 24.2 ± 2.7% in cirrhosis vs − 18.6 ± 2.2% in the healthy controls, *P* < 0.001). LAVI and E/e’ ratio were significantly higher in cirrhosis patients than in the normal controls, suggesting impaired LV diastolic function in cirrhosis patients. No significant difference in E/A ratio and deceleration time was found. LV diastolic function in cirrhosis patients could not be classified into one category in 15 patients (45.5%) when the 2016 guideline was applied [19]. In addition, the significant overlap of LA dimension, LAVI and even E/e’ ratio between cirrhosis patients and healthycontrols were observed (Fig. 1a, b, and c). Estimated pulmonary artery systolic pressure was significantly higher in patients with Child-Pugh class C than in patients with Child-Pugh class A/B (*P* = 0.040). As expected, QTc interval was significantly prolonged in cirrhosis patients (*P* < 0.001). Most ECG and echocardiographic parameters between Child-Pugh class A/B and Child-Pugh class C showed no statistical differences. A significant positive linear correlation was observed between QTc interval and Child-Pugh score (*r* = 0.388, *P* = 0.025).

**Table 2:** Baseline echocardiographic and electrocardiographic parameters

	Healthy controls	Cirrhosis	Child-Pugh class A/B	Child-Pugh class C	***P*** value^†^	***P*** value^*^
	***N*** = 17	***N*** = 33	***N*** = 10	***N*** = 23		
LVEF (%)	62.7 ± 5.6	66.0 ± 5.2	66.4 ± 3.5	65.8 ± 5.8	0.049	0.777
LV EDD (mm)	47.9 ± 4.0	48.0 ± 5.5	48.6 ± 4.8	47.7 ± 5.9	0.969	0.686
LV ESD (mm)	29.7 ± 4.3	28.0 ± 4.3	28.2 ± 3.4	28.0 ± 4.7	0.206	0.905
LV wall thickness (mm)	9.1 ± 0.9	8.9 ± 1.3	9.2 ± 1.3	8.8 ± 1.3	0.747	0.456
E/A ratio	0.9 ± 0.4	1.1 ± 0.4	1.1 ± 0.4	1.2 ± 0.5	0.165	0.431
Deceleration time (msec)	210.2 ± 30.2	212.3 ± 41.0	232.3 ± 39.6	203.2 ± 39.1	0.857	0.061
E/e’ ratio	8.6 ± 2.5	10.4 ± 2.5	10.8 ± 2.4	10.2 ± 2.5	0.018	0.431
Diastolic function					0.474	0.776
Normal	9 (52.9%)	17 (51.5%)	5 (50.0%)	12 (52.2%)		
Indeterminate	7 (41.2%)	15 (45.5%)	5 (50.0%)	10 (43.5%)		
Grade 1	1 (5.9%)	0 (0%)				
Grade 2	0 (0%)	1 (3.0%)	0 (0%)	1 (4.3%)		
LA dimension (mm)	38.2 ± 3.8	43.7 ± 7.7	47.1 ± 8.9	42.2 ± 6.7	0.008	0.094
LAVI (mL/m^2^)	39.8 ± 7.8	47.3 ± 11.2	45.6 ± 12.0	48.0 ± 11.0	0.020	0.565
Estimated PASP (mmHg)	32.1 ± 3.1	33.8 ± 5.3	31.0 ± 2.9	35.1 ± 5.7	0.348	0.040
GLS (%)	−18.6 ± 2.2	−24.2 ± 2.7	−25.2 ± 2.7	−23.8 ± 2.6	< 0.001	0.170
GCS (%)	−26.1 ± 4.7	−27.8 ± 5.1	−27.7 ± 5.1	−27.8 ± 5.2	0.254	0.950
Positive agitated saline test (n, %)	0 (0%)	22 (66.7%)	6 (60.0%)	16 (69.6%)	–	0.592
QTc interval (msec)	410.5 ± 8.6	470.3 ± 36.1	453.7 ± 19.9	477.6 ± 39.4	< 0.001	0.081

*LVEF* left ventricular ejection fraction, *EDD* end-diastolic diameter, *ESD* end-systolic diameter, *E* peak early diastolic mitral inflow velocity, *A* peak late diastolic transmitral peak velocity, *e’* early diastolic mitral annular velocity, *LA* left atrium, *LAVI* left atrial volume index, *PASP* pulmonary artery systolic pressure, *GLS* global longitudinal strain, *GCS* global circumferential strain, *QTc* corrected QT interval

^†^*P* value between normal and all liver cirrhosis groups

^*^P value between Child-Pugh class A/B and Child-Pugh class C

### CMR in cirrhosis patients

CMR-based hemodynamic parameters are shown in Table 3. LVEF was significantly higher in cirrhosis patients (*P* = 0.027), mainly driven by a larger EDV, rather than by a smaller ESV. Moreover, LV stroke volume (99.5 ± 29.3 vs 85.2 ± 15.1 mL, *P* = 0.025) and cardiac index (4.3 ± 1.1 vs 3.3 ± 0.7 L/min/m^2^, *P* = 0.002) were greater in cirrhosis patients, again supporting hyper-dynamic circulation in cirrhosis [2]. No differences in hemodynamic parameters between Child-Pugh class A/B and class C groups were found, although cardiac index tended to be higher in Child-Pugh class C group (*P* = 0.073). With Child- Pugh score as a continuous variable, a positive trend in relation to cardiac index was observed (*r* = 0.337, *P* = 0.06). There were no significant differences in terms of LV mass index and LV mass/LV EDV ratio between cirrhosis patients and the healthy control group.

**Table 3:** Baseline cardiovascular magnetic resonance parameters

	Healthy controls	Cirrhosis	Child-Pugh class A/B	Child-Pugh class C	***P*** value^†^	***P*** value^*^
	***N*** = 20	***N*** = 33	***N*** = 10	***N*** = 23		
LVEF (%)	63.4 ± 6.4	67.8 ± 6.9	66.1 ± 6.5	68.5 ± 7.1	0.027	0.368
LV EDV (mL)	135 ± 20	150 ± 47	153 ± 42	149 ± 50	0.101	0.811
LV ESV (mL)	51 ± 16	49 ± 21	52 ± 18	48 ± 23	0.752	0.604
Stroke volume (mL)	85 ± 15	100 ± 29	101 ± 28	99 ± 30	0.025	0.845
Cardiac index (L/min)	3.3 ± 0.7	4.3 ± 1.1	3.8 ± 1.0	4.5 ± 1.1	0.002	0.073
LV mass index (g/m^2^)	76.8 ± 13.0	70.7 ± 15.8	70.1 ± 13.0	71.0 ± 17.1	0.138	0.858
LV mass/LV-EDV ratio	0.9 ± 0.2	0.8 ± 0.3	0.8 ± 0.3	0.9 ± 0.2	0.126	0.875
Presence of LGE	0 (0%)	1 (3.0%)	0 (0%)	1 (4.3%)	0.432	0.503
Native T1 (msec)	1174 ± 65	1228 ± 79	1216 ± 60	1233 ± 87	0.001	0.584
ECV (%)	25.4 ± 1.9	31.6 ± 5.1	27.2 ± 3.4	33.6 ± 4.4	< 0.001	0.001

*LVEF* left ventricular ejection fraction, *EDV* end-diastolic volume, *ESV* end-systolic volume, *LGE* late gadolinium enhancement, *ECV* extracellular volume fraction

^†^*P* value between normal and all LC groups

^*^P value between Child-Pugh class A/B and Child-Pugh class C

**Fig. 2:**
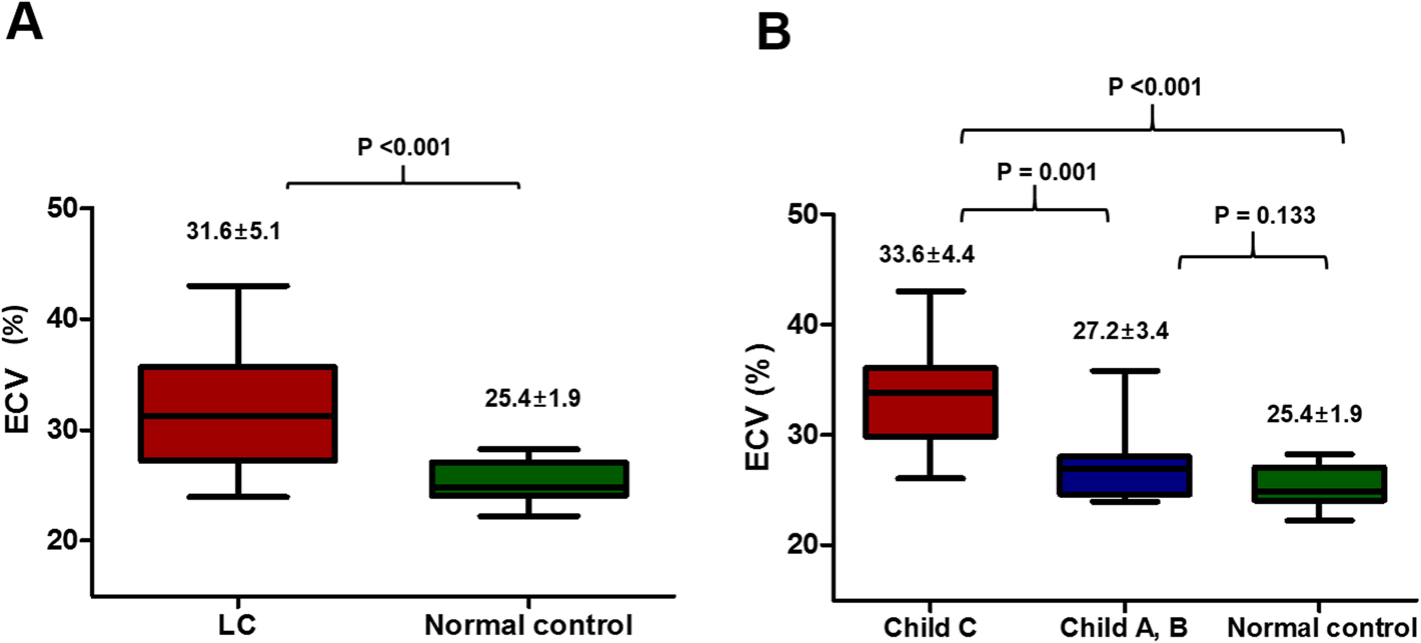
Diffuse myocardial fibrosis assessed by ECV in patients with liver cirrhosis (LC) and normal controls. **a** Extracellular volume (ECV) was significantly higher in cirrhosis patients. **b** Progressive increase in ECV was demonstrated from healthy controls to patients with Child-Pugh class C (*P* < 0.001)

**Fig. 3:**
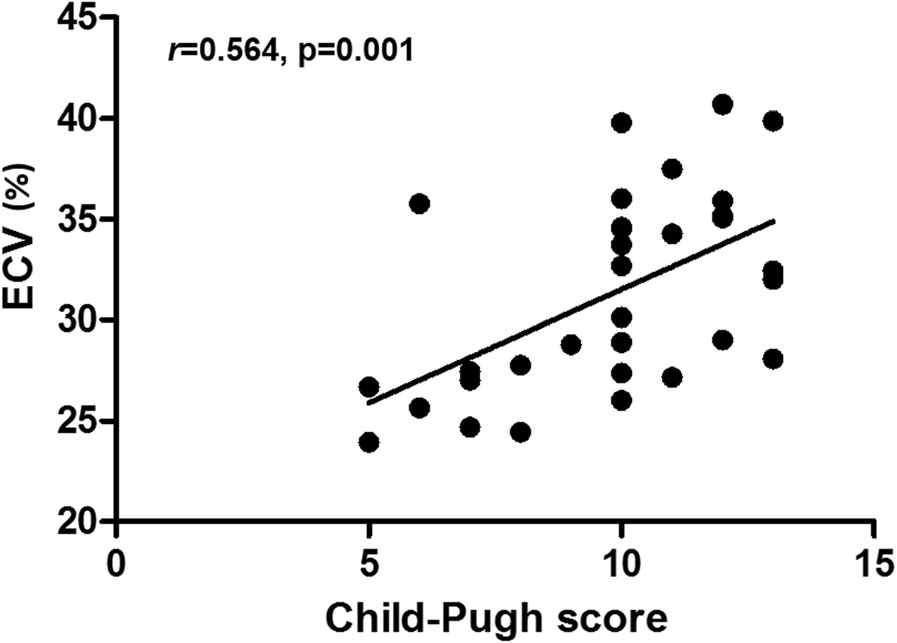
Relationship between the degree of diffuse myocardial fibrosis and cirrhosis severity. ECV denotes extracellular volume fraction

Focal subendocardial LGE was detected at the mid-anterior segment in one cirrhosis patient, whose coronary arteries were normal on invasive coronary angiography. No LGE was found in the healthy controls. Native T1 value of myocardium was significantly longer in patients with cirrhosis compared with healthy subjects (*P* = 0.001; Table 3). However, there was no statistically significant correlation between Child-Pugh score (as a continuous variable) and native T1 value in cirrhosis (*P* = 0.87). Cirrhosis patients had a significantly higher ECV (31.6 ± 5.1 vs 25.4 ± 1.9, *P* < 0.001; Table 3 and Fig. 2a), mainly driven by the cirrhosis patients with Child-Pugh class C (Table 3 and Fig. 2b). Besides, ECV was significantly higher in Child-Pugh class C than class A/B (33.6 ± 4.4 vs 27.2 ± 3.4, *P* = 0.001; Table 3 and Fig. 2b). Furthermore, a significant correlation between Child-Pugh score and ECV was noted (*r* = 0.564, *P* = 0.001; Fig. 3). Even after adjusting for cardiac index, the correlation between ECV and Child-Pugh score remained significant (*r* = 0.427, *P* = 0.019). The pre-transplant native T2 values measured in 6 patients at the last stage of this study were within normal range in all 6 patients (100%), whereas pre-transplant ECV was more than 30.0% in 4/6 patients (66.7%) (Table 4).

**Table 4:** Individual patient pre-transplant native T2 value and ECV

Patient	Pre-transplant native T2	Pre-transplant ECV
1	35.71	24.5%
2	44.41	34.6%
3	39.11	27.8%
4	44.72	32.6%
5	35.71	39.8%
6	36.48	43.0%

*ECV* extracellular volume fraction

### Changes in echocardiographic and CMR parameters 1 year after transplant

**Fig. 4:**
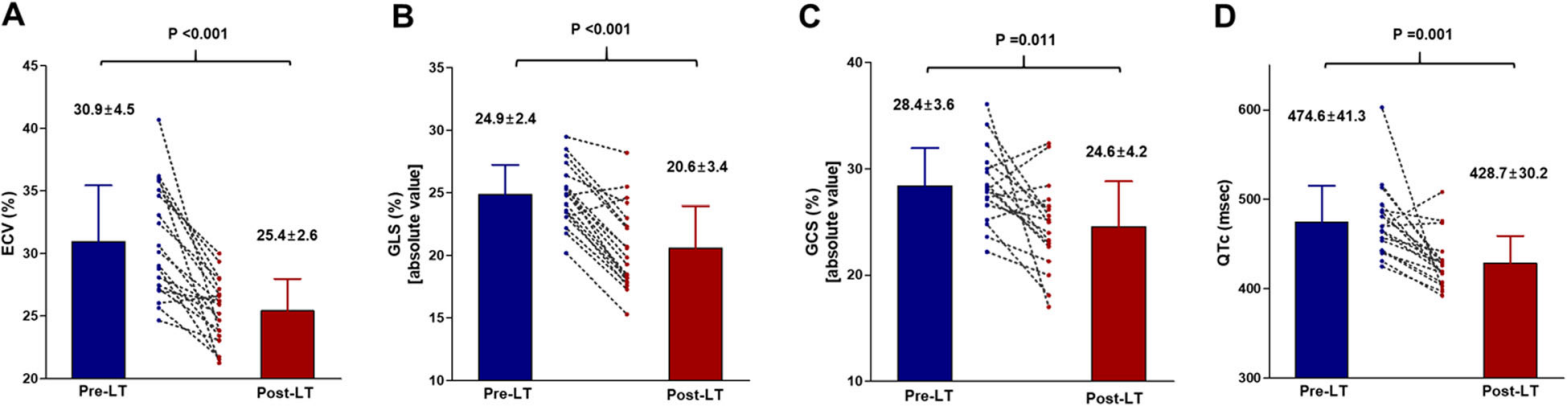
Liver transplantation-induced changes in ECV, global longitudinal strain (GLS), global circumferential strain (GCS) and electrocardiographic QTc interval. ECV, extracellular volume fraction; GLS, global longitudinal strain; GCS, circumferential strain; QTc, corrected QT

**Fig. 5:**
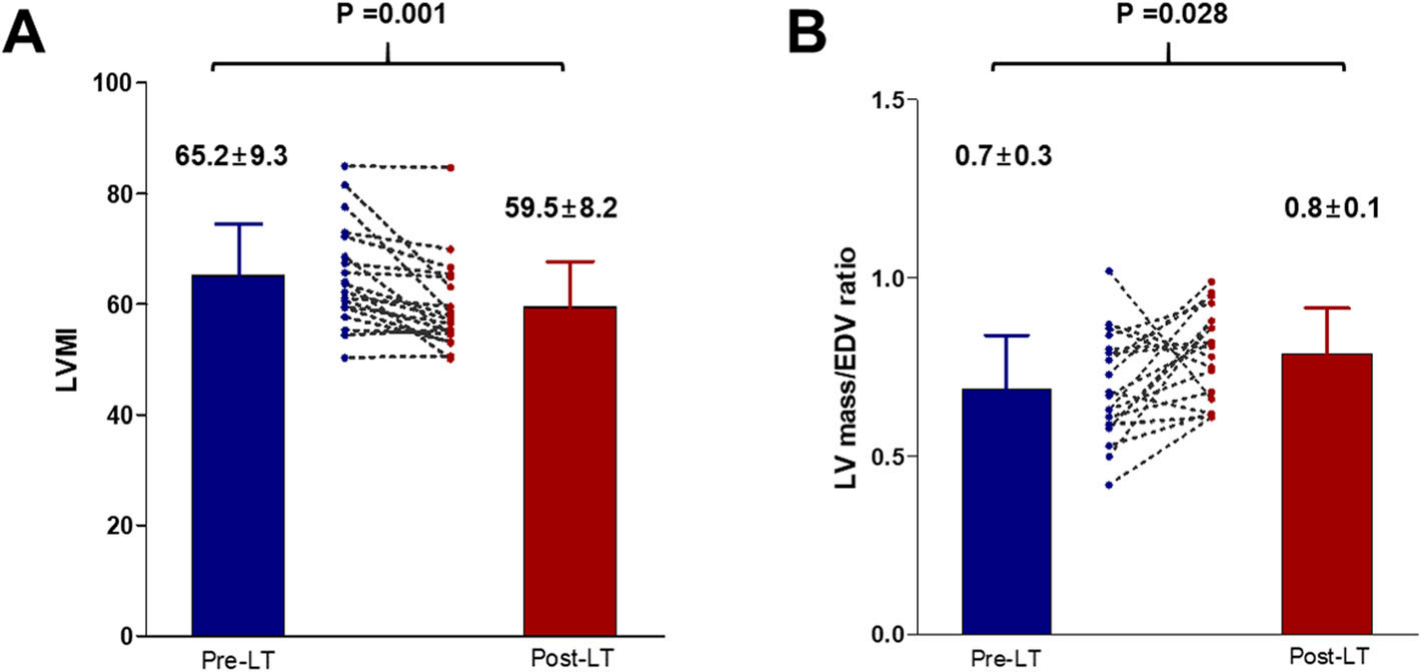
Changes of LV mass index (LVMI) and LV Mass / End-diastolic volume (EDV) ratio by cardiovascular magnetic resonance between pre- and post-liver transplantation. LVMI, left ventricular mass index; LVM/EDV, left ventricular mass/end-diastolic volume

A total of 28 patients underwent transplant, of which four (14.3%) died after transplantation. Three patients died of sepsis-associated heart failure within 6 months after transplant, and 1 patient died of heart failure 9 months after transplant. Patients who died after transplant were older, and had a lower pre-transplant CMR cardiac index (Additional file 1: Tables S1-S3). Of the 24 patients who survived transplant, 19 patients underwent echocardiography, ECG, and CMR 1-year post-transplant. Five patients refused follow-up examinations 1 year after transplant. Pre- and post- transplant ECG, echocardiographic and CMR parameters of the 19 patients were compared (Table 5 and Fig. 4). ECV showed a significant decrease 1 year after transplant (*P* < 0.001; Fig. 4a). Both LV end-diastolic diameter on echocardiography and LV EDV on CMR significantly decreased 1-year post- transplant (*P* = 0.003 and 0.001, respectively). Although CMR LVEF showed no significant changes 1-year post-transplant (*P* = 0.382), LV GLS (from − 24.9 ± 2.4% to − 20.6 ± 3.4%, *P* < 0.001; Fig. 4b) and GCS (from − 28.4 ± 3.6% to − 24.6 ± 4.2%, *P* = 0.011; Fig. 4c) on echocardiography significantly decreased 1 year after transplant. LV mass index by CMR showed a significant decrease 1 year after transplant, and LV concentricity by LV mass/LV EDV ratio showed a significant increase (Table 5 and Fig. 5). QTc interval also decreased (from 475 ± 41 msec to 429 ± 30 msec, *P* = 0.001; Fig. 4d). E/A ratio was significantly decreased 1-year post- transplant (from 1.2 ± 0.5 to 0.9 ± 0.3, *P* = 0.002). E/e’ ratio was significantly decreased, as well (11.0 ± 0.23 to 8.9 ± 2.9, *P* = 0.030).

**Table 5:** Changes in electrocardiographic, echocardiographic and CMR parameters in patients undergoing transplant

	Pre-transplant	1 year post-transplant	***P*** value
Echocardiography			
LVEF (%)	65.8 ± 5.0	62.5 ± 4.9	0.035
LV EDD (mm)	49.5 ± 4.7	46.0 ± 5.1	0.003
LV ESD (mm)	28.7 ± 3.9	27.9 ± 4.0	0.465
GLS (%)	−24.9 ± 2.4	−20.6 ± 3.4	< 0.001
GCS (%)	−28.4 ± 3.6	−24.6 ± 4.2	0.011
E/A ratio	1.18 ± 0.51	0.85 ± 0.27	0.002
E/e’ ratio	11.0 ± 0.23	8.9 ± 2.9	0.030
CMR			
LV EDV (mL)_	167 ± 48	130 ± 30	0.001
LV ESV (mL)	57 ± 22	48 ± 17	0.102
LVEF (%)	66.8 ± 6.8	65.1 ± 6.5	0.382
LV mass index (g/m^2^)	65.2 ± 9.3	59.5 ± 8.2	0.001
LV mass/LV-EDV ratio	0.7 ± 0.3	0.8 ± 0.1	0.028
Native T1 (msec)	1206 ± 72	1173 ± 73	0.121
ECV (%)	30.9 ± 4.5	25.4 ± 2.6	< 0.001
Electrocardiogram			
QTc interval (msec)	475 ± 41	429 ± 30	0.001

Abbreviations as in Tables 2 and 3

## Discussion

Hemodynamic adaptation in liver cirrhosis was first reported in 1953 [25]. Thereafter, cardiac dysfunction in cirrhosis has gained increasing attention, leading to coining the term ‘cirrhotic cardiomyopathy’. Functional and hemodynamic changes have been repeatedly described [4, 5, 26–28]; however, there has been a paucity of data regarding myocardial structural alterations in an in vivo setting. Here, we adopted CMR to demonstrate myocardial structural changes in transplant. CMR is best suited for myocardial tissue characterization in vivo, thanks to its unique LGE and T1 mapping techniques [12, 14, 22, 23]. Speckle-tracking echocardiography-derived GLS was also assessed to sensitively detect LV systolic functional changes, because it is known as the most sensitive and accurate index for systolic function [29, 30].

**Fig. 6:**
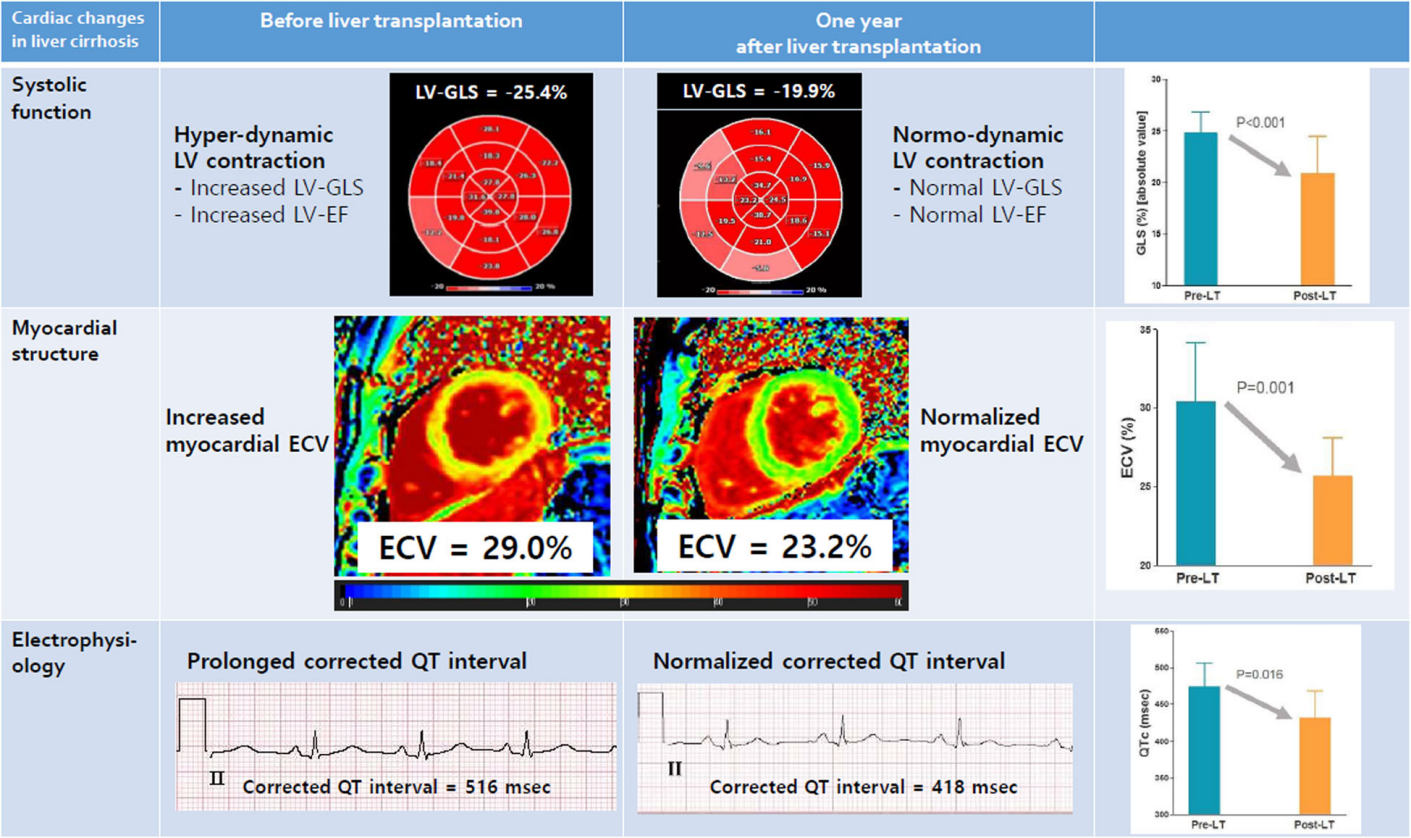
Myocardial structural, functional, and electrophysiological changes pre- and post-liver transplantation. ECV, extracellular volume fraction; GLS, left ventricular global longitudinal strain; QTc, corrected QT interval

The main findings of this study are summarized as follows and in Fig. 6: First, ECV was significantly increased in cirrhosis patients and showed a positive correlation with cirrhosis severity (assessed by Child-Pugh score; Fig. 3). Moreover, the QTc correlated with Child-Pugh score and ECV. These findings support that cirrhosis severity, myocardial structural changes, and myocardial electrical alterations are closely linked to each other. Second, assessment of resting LV diastolic function by echocardiography was impractical because almost half of cirrhosis patients (45.5%) could not be conclusively categorised based on the current guideline [19]. Finally, pre-transplant echocardiographic GLS was significantly augmented at rest, which recovered to the normal ranges 1 year after transplant. ECV and QTc normalization was also observed 1 year after transplant. Therefore, increased ECV and augmented GLS are considered two characteristic features of cirrhotic cardiomyopathy.

To the best of our knowledge, this is the first study to evaluate myocardial structural and functional characteristics before and after liver transplant using both CMR and speckle-tracking echocardiography in cirrhosis patients.

### Echocardiographic and electrophysiological components in the diagnosis of cirrhotic cardiomyopathy

Patients with cirrhosis experience a series of cardiovascular changes, including splanchnic arterial vasodilation with associated reduced systemic vascular resistance, neurohumoral axes dysfunction, and myocardial functional/electrophysiological changes. Apart from redistribution of circulating blood volume to the splanchnic bed, reduced systemic vascular resistance results in reduced central blood volume, subsequently leading to a *resting hyper-dynamic syndrome*. Thus, the resting hyper-dynamic state in cirrhosis by the hyper-contractile LV is considered an appropriate adaptive process to compensate for reduced central blood volume [1, 31]. This study also noted that LVEF was significantly greater in cirrhosis patients, which is supported by significantly increased echocardiographic GLS pre-transplant. No patient showed an LVEF < 55%, suggesting that the absolute number of resting LVEF itself is less clinically helpful to define cirrhotic cardiomyopathy. LV stain is more sensitive than LVEF to subtle changes in LV myocardial performance [29, 30]. We found that GCS in cirrhosis patients was not different from that in the normal controls, whereas GLS showed significant differences between the two groups. This finding suggests that GLS better reflects subtle changes in LV systolic performance, as suggested previously [29, 30, 32, 33].

LV diastolic dysfunction at rest was proposed as a diagnostic and supportive criterion for cirrhotic cardiomyopathy [2]. In this study, LAVI was greater in cirrhosis, indicating prolonged LV diastolic dysfunction in cirrhosis patients. Although E/e’ ratio was significantly higher in cirrhosis patients, it fell between 8 and 15, borderline values that cannot be used for clear differentiation between normal and increased LV filling pressure [34]. However, elevated LV filling pressure seemed to be present because E/A ratio was also > 1 in cirrhosis patients [35]. However, the significant overlap of LAVI and even E/e’ ratio between cirrhosis patients and the normal controls should be emphasized (Fig. 1b and c). Any cutoff value of LAVI or E/e’ ratio differentiating the two groups could not be suggested. Besides, LV diastolic function was not effectively categorised in approximately 50% of cirrhosis patients, suggesting that LV diastolic function assessment with resting echocardiography is neither practical nor sensitive approach for the diagnosis of cirrhotic cardiomyopathy. This observation makes sense given that overt structural changes in the LV are not a prerequisite for LV diastolic dysfunction development; simply, the aging process could lead to this change [35]. Although dobutamine or exercise stress echocardiography was suggested as alternative modalities to reveal subclinical LV systolic/diastolic dysfunction [28], a definite diagnosis of cirrhotic cardiomyopathy remains challenging [2, 6].

QTc prolongation was also suggested as a component of cirrhotic cardiomyopathy [27, 36]. Although the mechanisms are unclear, cirrhosis progression seems to be related to prolonged QTc, given that Child-Pugh score showed a positive correlation with QTc interval. Furthermore, we found that liver transplant normalized QTc prolongation 1 year after transplant (Fig. 4d), thereby showing a close relationship between the two. Thus, QTc prolongation should alert physicians for the possibility of cirrhotic cardiomyopathy; subsequently, efforts to identify other evidences of cirrhotic cardiomyopathy should be made.

### Myocardial structural alterations in cirrhosis

Data regarding myocardial histopathological changes in cirrhosis are limited [37]. Most of earlier investigations were conducted in an autopsy setting of alcoholic etiology, and thus whether the findings are attributable to alcoholic cardiomyopathy and whether similar findings could be anticipated in an in vivo setting remain to be established. CMR is best suited for this purpose with its unique ability to characterise myocardial tissues. LGE represents irreversible replacement fibrosis, while ECV represents reversible DMF [10, 13]. As myocardial fibrosis is associated with cardiac hypertrophy and a stiff, noncompliant LV [38], application of these two novel CMR techniques is clinically relevant for the indirect assessment of LV compliance or diastolic property. In this study, irreversible LGE was observed only in one cirrhosis patient. Absence of coronary artery disease on preoperative computed tomographic or invasive coronary angiography excluded the possibility of coronary disease in all patients. On the other hand, we observed a significant increment in ECV in cirrhosis, implying myocardial extracellular volume expansion. Additionally, we noted a significant relationship between ECV and cirrhosis severity. All of these findings support the earlier observation by Wiese et al. (i.e., ECV increased in cirrhosis patients) [8] and the concept of the heart-liver interaction [4, 39]. These observations highlight that an increased ECV in cirrhosis signifies real myocardial structural changes, thereby potentially representing a structural component of cirrhotic cardiomyopathy. This is of diagnostic value because current definition of cirrhotic cardiomyopathy includes only functional, hemodynamic, and electrocardiographic alterations, all of which are expected as secondary phenomena following structural alterations. Of note, native T1 value did not change significantly 1-year post-transplant, which was in clear contrast to ECV. This may be because native T1 value can be more affected than ECV by factors other than DMF. Thus, assessment of ECV is preferred to native T1 value to reliably detect myocardial changes in cirrhosis. The mechanism underlying an increased ECV is unclear. One possibility is that, in patients with cirrhosis, effective circulatory volume decreases, as portal hypertension progresses, and subsequently renin-angiotensin aldosterone system is stimulated [40]. Given that the renin-angiotensin aldosterone system is involved in chronic tissue damage and diverse organ dysfunction including myocardium [41], it is possible that DMF in cirrhosis is likely to be related to the activation of renin-angiotensin aldosterone system.

The interpretation that increased ECV in cirrhosis represents DMF may be debatable because increased intravascular volume can expand myocardial extracellular space, thereby resulting in an increased ECV. However, as cirrhosis progresses, redistribution of blood volume occurs with a decrease in the central circulation (i.e. central hypovolemia) and an increase of blood volume in splanchnic bed [1]. In this setting, increased myocardial intravascular volume is unlikely to occur. Moreover, the observation of a stepwise increase in ECV from healthy subjects, Child-Pugh class A/B, to Child-Pugh class C does not support the hypothesis that increased intravascular volume may be responsible for increased ECV. Pathologic data also suggests that DMF should be major myocardial structural changes in cirrhosis; Lunseth and colleagues found delicate DMF in 99 autopsied cirrhosis cases [9]. They described that the interposition of delicate fibrous tissues was frequently noted in the gap caused by transversely ruptured muscle fibers, however exudation or edema was present in only two cases [9]. Additionally, a previous study demonstrated that patients with myocardial edema showed decreased GLS regardless of LVEF [42]. Furthermore, we observed that the pre-transplant native T2 values assessed in 6 patients were all within normal ranges. Taken together, it is highly likely that increased ECV on CMR in cirrhosis patients predominantly represents DMF.

### Transplant-induced structural, functional, and electrocardiographic changes

Liver transplantation is the only curative treatment and has been believed to reverse structural, hemodynamic, and functional cardiac alterations in cirrhosis. However, only two prospective studies investigated transplant-induced cardiac changes. In the first study, 19 patients were evaluated with echocardiography before and 6 to 12 months after transplant. The authors found that transplant resulted in LV wall thickness regression, and a decrease in cardiac index and LVEF, but no change in LV diastolic function indexes [26]. The second study evaluated 40 patients with echocardiography before and 3 months after transplant. They observed LV diastolic function deterioration [43]. The main discrepancy between the two studies is regarding LV diastolic function, which implies practical difficulties in LV diastolic function assessment with echocardiography. Although echocardiography is currently the best non-invasive tool [19, 35], echocardiographic transmitral inflow velocities are difficult to interpret in the setting of LV load changes such as in transplantation [44]. Recognition of this limitation led us to focus on the hemodynamic, systolic functional, and structural changes in the LV. Interestingly, echocardiographic GLS in cirrhosis was significantly augmented pre-transplant, and normalized 1-year post-transplant. Furthermore, a change in GLS was accompanied by a significant ECV reduction and QTc normalization (Table 5).

Augmented GLS at rest in cirrhosis could be explained by the resting recruitment of LV contractile reserve to maintain hyper-dynamic circulation [31]. Resting recruitment of LV contractile reserve is potentially associated with a blunted inotropic response to physical or pharmacological stress that is previously described as a characteristic component of cirrhotic cardiomyopathy. Augmented GLS and increased ECV pre-transplant were normalized 1-year post-transplant, suggesting that GLS and ECV assessment could provide a good opportunity to sensitively and early detect transplant-induced minute changes in LV ultrastructure as well as systolic function. Interestingly, despite limited number of patients analysed, elevated ECV pre-transplant was fully reversible 1-year post-transplant in all cirrhosis patients. Thus, ECV estimation would not be required to confirm the restoration of myocardial health post-transplant.

We observed a stepwise increase in ECV from healthy subjects, Child-Pugh class A/B, to Child-Pugh class C, suggesting that ECV quantification can be clinically used to track myocardial health in relation to cirrhosis severity. Detection of this unique cardiomyopathy pre-transplant is clinically relevant and important given that up to 25% of cirrhosis patients were reported to experience cardiac death after transplant [1]. Of note, however, statistical significance was not achieved between the normal controls and cirrhosis patients with Child-Pugh class A/B, suggesting that early detection with CMR alone may be clinically challenging even if myocardial alteration begins at the early stage of cirrhosis. Further efforts should be made to more sensitively detect early stage of DMF in cirrhosis patients.

### Study limitations

Firstly, patients were enrolled in a prospective manner, but we had to exclude patients who could not undergo CMR, i.e. poor renal function or patients with acute clinical deterioration. Thus, our results could not be generalized to all terminally decompensated cirrhosis patients. Secondly, we did not demonstrate potential associations between CMR findings and long-term clinical outcomes. Finally, this study cannot provide definite clinical management directions to reduce the risk of cardiovascular events peri-operatively.

## Conclusions

Myocardial extracellular expansion with augmented resting LV systolic function was a characteristic finding in cirrhotic cardiomyopathy. This cardiac change was reversible 1 year after liver transplantation, suggesting that hepatic decompensation itself should be a culprit for the cause of the observed myocardial structural and functional changes. Thus, myocardial extracellular expansion represents a structural component of myocardial change in cirrhosis.

## Supplementary information

**Additional file 1: Table S1.** Comparison of baseline clinical characteristics of patients who died versus survived after transplantation **Table S2.** Comparison of baseline echocardiographic and electrocardiographic parameters of patients who died versus survived after transplantation. **Table S3.** Comparison of baseline cardiac magnetic resonance parameters of patients who died versus survived after transplantation.

## Data Availability

The datasets used and analyzed during the current study are available from the corresponding author on reasonable request.

## Abbreviations

CMR: Cardiovascular magnetic resonance
DMF: Diffuse myocardial fibrosis
ECG: Electrocardiogram
ECV: Extracellular volume fraction
EDV: End-diastolic volume
ESV: End-systolic volume
GCS: Global circumferential strain
GLS: Global longitudinal strain
LA: Left atrium/left atrial
LAVI: Left atrial volume index
LGE: Late gadolinium enhancement
LV: Left ventricle/left ventricular
LVEF: Left ventricular ejection fraction
LVMI: Left ventricular mass index
QTc: Corrected QT interval
