# Supplementary material for: Myocardial structural and functional changes in patients with liver cirrhosis awaiting liver transplantation: a comprehensive cardiovascular magnetic resonance and echocardiographic study
Source: J Cardiovasc Magn Reson. 2020 Apr 23;22:25. doi: 10.1186/s12968-020-00622-2 (PMC7178724; doi:10.1186/s12968-020-00622-2)
Supplement: Supplementary file 1 — Additional file 1: Table S1. Comparison of baseline clinical characteristics of patients who died versus survived after transplantation Table S2. Comparison of baseline echocardiographic and electrocardiographic parameters of patients who died versus survived after transplantation. Table S3. Comparison of baseline cardiac magnetic resonance parameters of patients who died versus survived after transplantation. [file 12968_2020_622_MOESM1_ESM.docx]

**Supplementary Materials**

**Myocardial Structural and Functional Changes**

**in Patients with Liver Cirrhosis Awaiting Liver Transplantation**

**; A Comprehensive Cardiac Magnetic Resonance and Echocardiography Study**

Hyue Mee Kim, MD^a,b^, *Hyung-Kwan Kim, MD, PhD^a^, *Jeong-Hoon Lee, MD, PhD^c^,

Yun Bin Lee, MD, PhD^c^, Eun-Ah Park, MD, PhD^d^, Jun-Bean Park, MD, PhD^a^,

Seung-Pyo Lee, MD, PhD^a^, Yoon Jun Kim, MD, PhD^c^, Yong-Jin Kim, MD, PhD^a^,

Jung-Hwan Yoon, MD, PhD^c^, Dae-Won Sohn, MD, PhD^a^,

^a^Cardiovascular Center, Department of Internal Medicine, Seoul National University Hospital, Seoul, South Korea

^b^Division of Cardiology, Heart Stroke Vascular Center, Mediplex Sejong Hospital, Incheon, South Korea

^c^Department of Internal Medicine and Liver Research Institute, Seoul National University College of Medicine, Seoul, South Korea

^d^Department of Radiology, Seoul National University Hospital, Seoul, South Korea

**Running title:** Myocardial structural and functional changes in LC

**Addresses for correspondence:**

Hyung-Kwan Kim, MD, PhD

Professor / Director of Cardiac Diagnostic Test Unit

Section of Cardiovascular Imaging, Division of Cardiology

Department of Internal Medicine and Cardiovascular Center

Seoul National University Hospital

103 Daehak-ro, Jongno-gu, Seoul, 03080, South Korea

Tel.: +02-2072-0243 / Fax: +82-2-2072-2577

E-mail address: [cardiman73@gmail.com](mailto:cardiman73@gmail.com) or [hkkim73@snu.ac.kr](mailto:hkkim73@snu.ac.kr)

or

Jeong-Hoon Lee, MD, PhD

Associate Professor,

Division of Gastroenterology, Department of Internal Medicine

Seoul National University Hospital

103 Daehak-ro, Jongno-gu, Seoul, 03080, South Korea

Tel.: +02-2072-2228 / Fax: +82-2-743-6701

E-mail address: [pindra@empal.com](mailto:pindra@empal.com)

**Table S1.** **Comparison of** **baseline clinical characteristics of patients who died versus survived after LT.**

|  | **Died** | **Survived** | **P value** |
| --- | --- | --- | --- |
|  | **N=4** | **N=24** |  |
| Age (years) | 68.3±9.7 | 54.5±9.3 | 0.007 |
| Male (n, %) | 3 (75.0%) | 18 (75.0%) | 1.000 |
| Systolic blood pressure (mmHg) | 114.5±8.4 | 114.6±15.1 | 0.825 |
| Diastolic blood pressure (mmHg) | 59.5±2.6 | 68.0±12.6 | 0.164 |
| Heart rate (/min) | 78.0±23.7 | 73.9±15.0 | 0.924 |
| **Cirrhosis etiology (n, %)** |  |  | 0.459 |
| Viral | 2 (50.0%) | 16 (66.7%) |  |
| Alcoholic | 1 (25.0%) | 5 (20.8%) |  |
| Autoimmune hepatitis | 0 (0%) | 2 (8.3%) |  |
| Cryptogenic | 1 (25.0%) | 1 (4.2%) |  |
| Child-Pugh score | 9.0±2.7 | 9.8±2.4 | 0.590 |
| MELD score | 19.5±10.8 | 18.9±7.3 | 0.975 |
| **Underlying diseases (n, %)** |  |  |  |
| Hypertension | 2 (50.0%) | 5 (20.8%) | 0.212 |
| Diabetes mellitus | 1 (25.0%) | 6 (25.0%) | 1.000 |
| **Medication (n, %)** |  |  |  |
| Beta-blockers | 1 (25.0%) | 7 (29.2%) | 0.864 |
| Diuretics | 1 (25.0%) | 11 (45.8%) | 0.436 |
| ACEI/ARB | 1 (25.0%) | 2 (8.3%) | 0.318 |
| **Laboratory examination** |  |  |  |
| Hemoglobin (g/dL) | 11.1±2.5 | 10.5±1.5 | 0.547 |
| Creatinine (mg/dL) | 1.3±0.6 | 0.8±0.3 | 0.059 |
| Bilirubin (mg/dL) | 5.4±6.2 | 8.5±9.4 | 0.505 |
| Albumin (g/dL) | 3.3±0.6 | 2.9±0.4 | 0.144 |
| PT (INR) | 1.4±0.4 | 2.0±1.1 | 0.126 |

Values are shown as number (%) or mean ± standard deviation

LT, liver transplantation; MELD, the model for end stage liver disease; ACEI, angiotensin-converting enzyme inhibitors; ARB, angiotensin II receptor blockers; PT, prothrombin time; INR, international normalized ratio

**Table S2**. **Comparison of** **baseline echocardiographic and electrocardiographic parameters of patients who died versus survived after LT.**

|  | **Died** | **Survived** | | **P value** |
| --- | --- | --- | --- | --- |
|  | **N=4** | **N=24** | |  |
| LV-EF (%) | 65.5±3.7 | | 65.1±5.3 | 0.776 |
| LV-EDD (mm) | 47.5±3.7 | | 48.7±5.3 | 0.547 |
| LV-ESD (mm) | 28.0±3.6 | | 28.8±4.0 | 0.681 |
| LV wall thickness (mm) | 9.5±1.3 | | 9.0±1.3 | 0.465 |
| E/A ratio | 0.7±0.1 | | 1.2±0.4 | 0.002 |
| DT (msec) | 230.8±15.9 | | 214.4±42.7 | 0.303 |
| E/e’ ratio | 10.3±0.4 | | 10.6±2.4 | 0.975 |
| Diastolic function |  | |  | 0.456 |
| Normal | 1 (25.0%) | | 13 (54.2%) |  |
| Indeterminate | 3 (75.0%) | | 10 (41.7%) |  |
| Grade 1 | 0 (0%) | | 0 (0%) |  |
| Grade 2 | 0 (0%) | | 1 (4.2%) |  |
| LA dimension (mm) | 46.0±7.4 | | 44.5±8.0 | 0.505 |
| LAVI (mL/m^2^) | 50.4±14.2 | | 48.7±10.4 | 0.825 |
| Estimated PASP (mmHg) | 35.0±6.5 | | 33.2±5.4 | 0.681 |
| LV-GLS (%) | -21.9±0.8 | | -24.5±2.8 | 0.012 |
| LV-GCS (%) | -25.8±5.4 | | -27.4±4.9 | 0.560 |
| QTc interval (msec) | 454.8±15.3 | | 470.1±38.2 | 0.505 |

LT, liver transplantation; LV, left ventricle; EF, ejection fraction; EDD, end-diastolic diameter; ESD, end-systolic diameter; E, early diastolic mitral inflow velocity; A, late diastolic mitral inflow velocity; DT, deceleration time; e’, early diastolic mitral annular velocity; LA, left atrium; LAVI, left atrial volume index; PASP, pulmonary artery systolic pressure; GLS, global longitudinal strain; GCS, global circumferential strain; QTc, corrected QT

**Table S3**. **Comparison of baseline cardiac magnetic resonance parameters of patients who died versus survived after LT.**

|  | **Died** | **Survived** | **P value*** |
| --- | --- | --- | --- |
|  | **N=4** | **N=24** |  |
| LV-EF (%) | 66.0±5.6 | 67.4±7.2 | 0.776 |
| LV-EDV (mL) | 118.6±55.5 | 159.2±46.5 | 0.186 |
| LV-ESV (mL) | 42.1±25.4 | 53.0±21.6 | 0.322 |
| Stroke volume (mL) | 76.5±30.4 | 104.4±28.3 | 0.147 |
| Cardiac index (L/min/m^2^) | 3.1±1.2 | 4.5±1.0 | 0.041 |
| LV mass index (g/m^2^) | 76.7±21.8 | 69.7±14.5 | 0.465 |
| LV mass/LV-EDV ratio | 1.2±0.3 | 0.8±0.2 | 0.015 |
| Presence of LGE | 0 (0%) | 1 (4.2%) | 0.678 |
| Native T1 (msec) | 1263.2±68.1 | 1221.6±66.0 | 0.291 |
| ECV (%) | 30.9±8.2 | 31.2±5.0 | 0.799 |

LT, liver transplantation; LC, liver cirrhosis; LV, left ventricle; EF, ejection fraction; LV-ED(S)V, left ventricular end-diastolic (end-systolic) volume; LGE, late gadolinium enhancement; ECV, extracellular volume fraction.
